# Signal Processing for Parametric Acoustic Sources Applied to Underwater Communication †

**DOI:** 10.3390/s20205878

**Published:** 2020-10-17

**Authors:** María Campo-Valera, Ivan Felis-Enguix, Isidro Villó-Pérez

**Affiliations:** 1 Departamento de Electrónica, Tecnología de Computadores y Proyectos, Universidad Politécnica de Cartagena (UPCT), 30202 Cartagena, Murcia, Spain; maria.campo@edu.upct.es (M.C.-V.); isidro.villo@upct.es (I.V.-P.); 2 Laboratorio de Hidroacústica (LHA), Centro Tecnológico Naval y del Mar (CTN), Ctra El Estrecho-Lobosillo, Km. 2, 30320 Fuente Álamo, Murcia, Spain

**Keywords:** underwater acoustic communication, non-linear acoustic, parametric effect, modulation techniques, acoustic propagation

## Abstract

For years, in the field of underwater acoustics, a line of research with special relevance for applications of environmental monitoring and maritime security has been developed that explores the possibilities of non-linear phenomena of sound propagation, especially referring to the so-called parametric effect or self-modulation. This article shows the results of using a new modulation technique based on sine-sweep signals, compared to classical modulations (FSK and PSK). For each of these modulations, a series of 16-bit strings of information with different frequencies and durations have been performed, with the same 200 kHz carrier wave. All of them have been tested in the Hydroacoustic Laboratory of the CTN and, through the application of cross-correlation processing, the limitations and improvements of this novel processing technique have been evaluated. This allows reaching better limits in discrimination of bits and signal-to-noise ratio used in underwater parametric acoustic communications.

## 1. Introduction

The study of parametric generation has generated great interest since Westervelt published his article in 1963 [1]. Subsequently, it has become popular in the field of underwater acoustics as regards the processing of parametric array signals [2,3,4]. These parametric acoustic sources are gradually being used in detection sonars and communication sonars in shallow water [5,6,7,8,9,10,11].

Among the aspects of parametric underwater acoustic communications, on one hand, it is necessary to reduce the multipath effect, which makes it possible to simplify the analysis of the signal sent when, for example, a system made up of ROV underwater vehicles is operated remotely from an emitting source and, on the other hand, reduce interference between users by achieving a larger-scale operation.

Parametric underwater acoustic communications use the non-linear effects that are generated in the underwater channel, for data transmission. When a high intensity and high-frequency signal is transmitted, a low-frequency wave (corresponding to the parametric difference frequency $\left(f_{d}=f_{m1}-f_{m2}\right)$ is formed in the submarine channel due to intermodulations on the transmitted wave. Using modulation techniques if appropriate, the transmitted signal information can be retrieved from the non-linearly generated waveform. The generated low-frequency waveform has a large bandwidth and can propagate over long distances. However, the most important benefit is the fact that achieves high directivity at these low frequencies using physically small transducers, thus these parametric communications promote a promising approach to data transmission on long-range acoustic submarine channels.

### 1.1. Background

In 1965, Berktay and Smith [12] introduced their well-known theory of self-demodulation of the medium, which consists in the emission of an amplitude-modulated carrier frequency wave (primary beam) with another lower frequency (modulator signal). The medium that by non-linear effects is responsible for demodulating the emitted wave gives rise to new frequencies, among them the difference frequency. This theory has been widely used in the design of parametric sonars.

The theory of the emission of a parametric broadband signal assuming that the acoustic signal propagates in flat waves deduced by Berktay, which corresponds to the pressure of a wave that travels along the *x* axis in time *t* and distance *r*, is represented as follows:

(1) $$p\left(t,r\right)=Pe^{-\alpha_{p}r}f\left(t-\frac{r}{c}\right)\cos\left[2\pi f_{p}\left(t-\frac{r}{c}\right)\right]$$

where *P* is the amplitude of the sound pressure, $\alpha_{p}$ is the absorption coefficient of the carrier signal, $\cos\left(2\pi f_{p}t\right),f\left(t-r/c\right)$ is the envelope of the modulation defined by the modulating wave and *c* is the speed of the sound in the water. From (1), the acoustic pressure is derived for the difference frequency in the axial axis and at a distance *x* in the far-field, defined as:

(2) $$p_{s}\left(t,r\right)=-A\frac{\partial^{2}}{\partial t^{2}}\left[E^{2}\left(t\right)\right]$$

where *A* is a constant related to the amplitude, the absorption of the medium and the vibrating surface of the transducer and E(t) is the envelope of the function.

The Berktay and Smith [12] equation is a good approximation for directional beams close to the axis and states that the demodulated signal (low frequency or parametric difference frequency) is proportional to the second derivative in time of the square envelope of the carrier of the modulated signal in amplitude. This acoustic model is the most widely used in preprocessing methods for parametric sources [13]. The characteristics of the self-demodulated wave depend on the primary waves and the amplitude of the difference frequency is proportional to the square of the carrier frequency.

Equation (2) provides the modulation schemes that are commonly used in underwater acoustic communications in parametric arrays.

### 1.2. Aproaches

In the first instance, some of the digital modulation techniques are studied, among which its representation is possible parametrically through the sending and reception of a string of bits (ones and zeros), and they are compared with each other regarding their detection capacity. including the bit error. Finally, the advantages of parametric acoustic communication with respect to improvements in multipath propagation are compared with a conventional acoustic source.

## 2. Modulation Techniques

This study aims to study different digital modulation techniques to transmit information about a given carrier wave, normally analogue of the sine type. These techniques allow better use of the communication channel, which makes it possible to transmit more information simultaneously, protecting it from possible interferences using non-linear signals to be applied in acoustic communications.

Modular consisting of making a parameter of the carrier wave change its value according to the variations of the modulating signal (amplitude, frequency, and phase), which corresponds to the information to be transmitted.

The modulations used in this study consist of analogue carriers modulated through a digital modulating signal, where the most basic binary symbol is the digit (1 or 0) [2].

As not all the digital modulations used in the linear range can be used, since, to be able to generate them parametrically, a double integral process must be carried out, which does not always make it possible to use them in all modulations. of which its obtaining is possible. From Berktay’s Equation (2) it is assumed that the carrier signal is an amplitude modulated waveform expressed as:

(3) $$x\left(t\right)=A_{p}E\left(t\right)\cdot \sin\left(2\pi f_{p}t\right)$$

where $A_{p}$ is the amplitude of the carrier signal, E(t) is the envelope of the modulation defined by the modulator wave, and $\sin(2\pi f_{p}t)$ is the frequency of the carrier signal. We have that the modulated signal x(t) consists of the result of the product of the carrier signal with the modulator.

Among the modulations studied in question in this article, a set of signals for FSK, PSK, and a modulation using frequency sweeps (Sine-sweeps) are shown, which will be explained later. Next, the typical schemes of these digital communication modulations that use the parametric generation phenomenon are presented.

### 2.1. FSK Modulation

It is a modulation that uses an analogue carrier frequency and the modulator is a binary signal z(t) that has amplitude 1 V and −1 V for bit 1 and 0 respectively [14].

These signals can be obtained parametrically using a modulated signal whose modulation FSK corresponds, alternatively, to two signals of modulating frequencies $f_{m1}$ and $f_{m2}$, that must be half of the frequencies associated with each one of the bits that we want to receive, which is, properly, another parametric type signal [7]. Thus, through this non-linear technique, by modulating a carrier with an FSK, another FSK of twice the frequency is obtained. Depending on the bit to send, its mathematical representation is given as follows:

(4) $$\begin{array}{c} z_{\mathrm{FSK}[bit1]}\left(t\right)=A_{p}\sin\left[(2\pi f_{m1}/2)t\right] \end{array}$$

(5) $$\begin{array}{c} z_{\mathrm{FSK}[bit0]}\left(t\right)=A_{p}\sin\left[(2\pi f_{m2}/2)t\right] \end{array}$$

**Figure 1:**
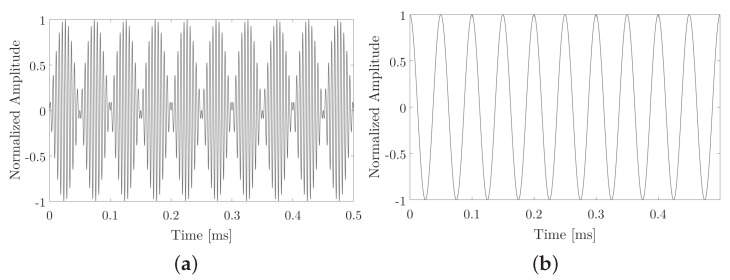
Bit 1 example. (**a**) FSK modulation. (**b**) FSK type signals that want to obtain parametrically.

In the following example, we have two modulations linked to the half of the desired parametric which, for this case, is 5 kHz for bit 1 and 10 kHz for bit 0, with 500 μs duration for each bit and an $f_{p}$ at 200 kHz. The following Figure 1, presents the signals that were used to encode bit 1. These consist of one parametric tone expected at the frequencies of 10 kHz.

### 2.2. PSK Modulation

In this modulation, the phase of the carrier frequency is shifted according to the value of the information data bit (binary modulating signal). Where bit 1 corresponds to 0° and bit 0 to 180°. The mathematical representation if multiplied by a logical 1 or logical 0 is given below.

Parametrically, these signals are obtained by using as a modulating signal for bit 1, $f_{m1}$ a certain frequency that, as will be seen later, different frequency values were used for this modulator, and for bit 0 the same frequency plus a phase shift of 180° and using for all cases a carrier frequency of 200 kHz. Thus, we obtain.

(6) $$\begin{array}{c} z_{\mathrm{PSK}[bit1]}\left(t\right)=A_{p}\sin\left(2\pi f_{m1}/2\right)t) \end{array}$$

(7) $$\begin{array}{c} z_{\mathrm{PSK}[bit0]}\left(t\right)=A_{p}\sin\left(2\pi f_{m1}/2\right)t+\pi ) \end{array}$$

**Figure 2:**
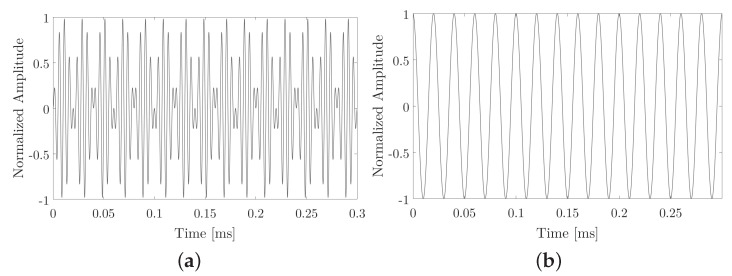
Bit 1 example. (**a**) PSK modulation. (**b**) PSK type signals that want to obtain parametrically, in this case, this signal is also equal to bit 0.

Figure 2, shows an example of a PSK with an expected frequency of 50 kHz at 300 μs and $f_{p}$ of 200 kHz.

### 2.3. Sine-Sweep Modulation

As we have seen, the modulation techniques are based on the concatenation of analogue sine-type signals to obtain parametric digital modulations with a digital modulating signal and analogue carrier signals. Therefore, a type of modulation has been developed that consists of the sequence of several sine-like signals; that is, sinusoidal sweeps, where bit 1 has an upward sweep and for bit 0 a downward sweep. This modulation technique is explained below.

This modulation consists of changing the amplitude of the carrier signal as a function of the modulating signal (information). In this work, to improve the behaviour of the previous modulations, two sine-sweeps are used as a modulating signal, upward (bit 1) and other downward (bit 0). The expression that defines the modulating signal is expressed as:

(8) $$\begin{array}{c} z_{\text{sine-sweep}}\left(t\right)=\sin\left[2\pi \left(\frac{|f_{m2}-f_{m1}|}{T}\cdot t+f_{m1}\right)t\right] \end{array}$$

where $f_{m1}$ and $f_{m2}$ are the initial and final frequency of the sweep, respectively and *T* is the total sine sweep duration.

**Figure 3:**
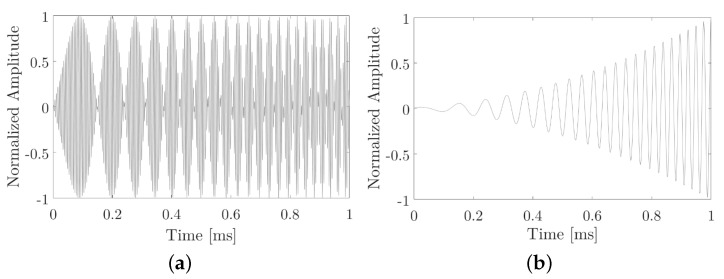
Bit 1 example. (**a**) sine-sweep modulation. (**b**) Parametric signals expected when sending sine-sweep modulation.

Figure 3, shows an example of the one-bit signal, corresponds to an expected sweep from 10 to 50 kHz at 1000 μs with 200 kHz $f_{p}$. The expected parametric signal is presented when sending with sine-sweep modulation.

## 3. Experimental Set-Up

**Figure 4:**
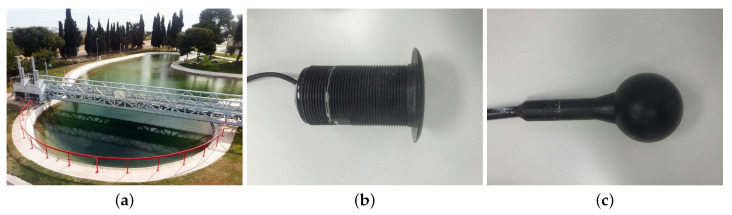
Location where the experimental measurements were made. (**a**) Lake; (**b**) Airmar P19 emitter; (**c**) ITC 1032 receiver.

The measurements were carried out at the Centro Tecnológico Naval y del Mar (CTN) in agreement with the Universidad Politécnica de Cartagena in Murcia, Spain in a lake of tapered shape with a 10 m depth and a diameter of 20 m. The next Figure 4 is a picture of the experimental setup.

An ITC 1032 transducer was used as a receiver with a receiving sensitivity of −194 dB re 1 V/μPa, without much variation at the resonance frequency region at 33 kHz and below, and thus was quite sensitive to the low frequencies willing to be detected. The Airmar P19 plane transducer was chosen as acoustic transmitter. In this study, the carrier frequency used in all signals is 200 kHz, with a sampling frequency of 20 Ms/s.

## 4. Detection Capacity and Results

Cross-correlation is a mathematical operation that is used to measure the degree of similarity between two signals in order to extract certain desired information. Furthermore, if there is some similarity in the waveforms x(t) and y(t) [3]. The signals under study are worked in discrete time, whereby the correlation between the two signals x[n] and y[n] with several samples *N*, is announced by the following expression:

(9) $$r_{xy}\left[l\right]=\sum_{n=1}^{N}x\left[n\right]\;y\left[n+l\right]$$

It can be assumed that the signal digitized by the emitting transducer is x[n] and the digitized signal from the hydrophone is y[n], where l=1,2,…,n, are the number of samples in which y[n] is delayed. Thus, the cross-correlation function is determined for several values *l* and the estimate of the delay time will be between the values of *l* at which the correlation function is maximum.

For the correct detection of the bits, the maximum peak obtained by correlating the received signal with the emitted signal establishes the arrival time of the received signal. Knowing the speed of sound propagation in the medium where the signal is transmitted, the distance between emission and reception is determined. To expand more check reference [4].

In this study, the detection of the correlation peaks of the received signal is interesting, however, these signals will be influenced by the reflections that can intervene in the amplitude and detection of the direct signal, therefore, through the study Cross-correlation makes it easier and more efficient to discern the direct signal from reflections, especially for signals with a higher bandwidth (narrower correlation peak). With all of the above, a correlation analysis of an experimental measurement of a received parametric sweep is performed both ascending (bit 1) and descending (bit 0), and they are correlated with the envelope E(t) (low parametric frequency) ascending and descending respectively.

The objective of this study is to detect by correlation bit 1 and bit 0. Therefore, if an upward sweep (bit 1) is correlated with its upward envelope, it is that such an event is detected. Conversely, if the upward sweep (bit 1) is correlated with the falling envelope; no event should be detected. The same should be expected for bit 0 detection, correlating the received sweep downward with the envelope downward. This analysis is presented below where the received signal is for bit 1 and bit 0, correlating it with the envelope (bit 1 and bit 0).

**Figure 5:**
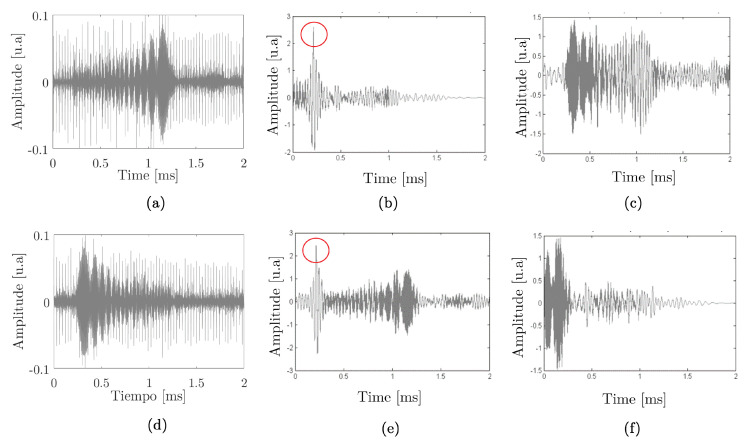
Detection by correlation. (**a**) Bit 1 received; (**b**) correlation of bit 1 received with bit 1 sent; (**c**) correlation of bit 1 received with bit 0 sent; (**d**) bit 0 received; (**e**) correlation of bit 0 received with bit 0 sent; (**f**) correlation of bit 0 received with bit 1 sent.

In Figure 5a,d, presented the upward and downward sine-sweep respectively of the received signal. Figure 5b, when correlating the received upward sweep (bit 1) with the envelope (expected parametric signal) of bit 1, a correlation peak in the expected time of 0.2 ms is observed for the expected distance between transducers (30 cm); we will call this “true bit”, while Figure 5c in case of the correlation with the envelope of bit 0, shows that there is no correlation maximum; we will call false bit. Regarding Figure 5e, the correlation of the received descending sweep (bit 0) with the envelope of bit 0 shows an expected correlation maximum of 0.2 ms, unlike Figure 5f; (true bit) which, when correlated with bit 1, no correlation peak is required (false bit). With this, the goodness of cross-correlation as a technique for detecting digital signals, starting from sweeps parametric signals, becomes evident.

Next, the results of the study of a 16-bit string organized as follows [01101111 01101011] are presented below, corresponding to the word “ok”. Detection and discrimination analyzes of this bit string are presented.

### 4.1. Cross-Correlation Detection of FSK Modulation

**Figure 6:**
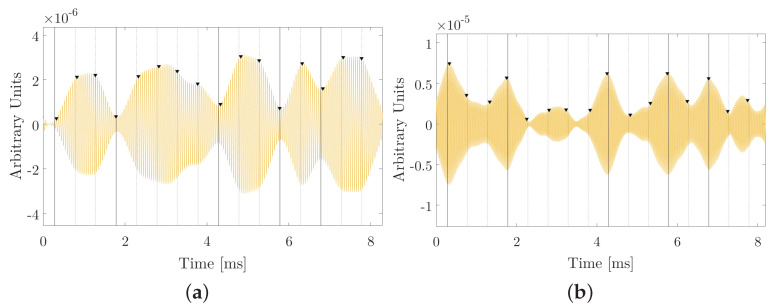
Correlation analysis for FSK. (**a**) Cross-correlations between the received signal and the expected 1 bit de 10 kHz, (**b**) and between the received signal and the expected 0 bit 20 kHz a 500 μs duration per bit.

Expected frequencies of each bit 1 and 0 are 10 and 20 kHz, respectively. These signals are correlated with each of the expected bits, obtaining the cross-correlations shown in Figure 6

It is observed that the correlation peaks are quite wide (in the order of the duration of each bit, approximately) because this type of modulation is, in essence, a pure tone that changes in frequency. correlations with narrowband signals are characterized by not being very efficient in their detection and temporal discrimination.

The average of the amplitudes of bits 1 and 0 correctly detected is $0.02410^{-4}$ and $0.061\times 10^{-4}$ arbitrary units, respectively. The average of the false bits is $0.007\times 10^{-4}$ and $0.014\times 10^{-4}$. The quotient between the correctly detected bits with respect to the false ones can give us an estimate of the error bit, as will be seen in the next section. Amplitude ratio between the true bits 1 with respect to the false ones is 3.43, and amplitude quotient between true 0 bits with respect to false bits is 4.35. These data give us an estimate of the SNR for this modulation.

### 4.2. Detection by Cross-Correlation of PSK Modulation

**Figure 7:**
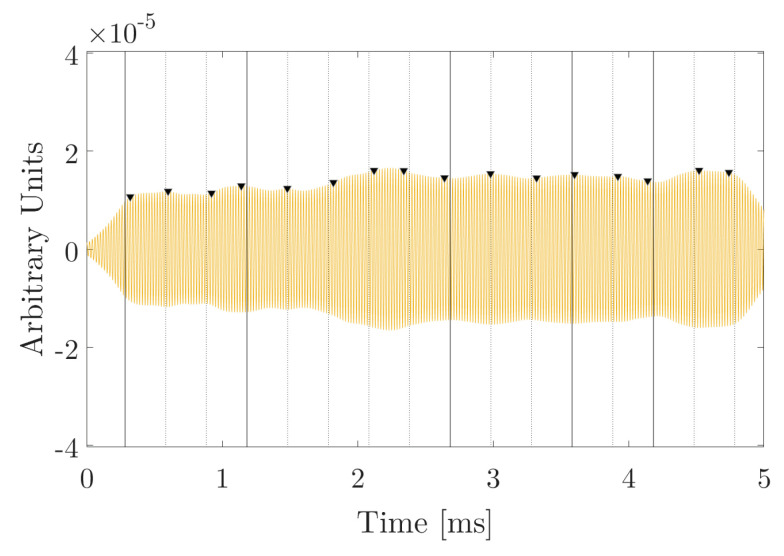
Correlation analysis. Cross-correlations between the received signal and the expected 1 bit of 50 kHz, at 300 μs duration per bit. This received signal is the same for bit 0.

In Figure 7, we have an example for one of the PSK signals measured in the pool, with a $f_{p}$ of 200 kHz and an expected frequency of 50 kHz at 300 μs. As observed, there is no differentiation between the received bits (1 and 0) at the time of their detection, since there is an amplitude value for bit 1 and 0 of $0.14\times 10^{-4}$ and $0.13\times 10^{-4}$ respectively, while the amplitudes of the false bits correspond to $0.13\times 10^{-4}$ and $0.14\times 10^{-4}$ respectively.

Amplitude ratio between the true bits 1 with respect to the false ones is 1.08, and amplitude quotient between true 0 bits with respect to false bits is 0.93. These data give us an estimate of the SNR for this modulation.

Because the expected signal for bit 1 and bit 0 is the same Figure 7, the received signal is the same for both bits.

In this study in question, this type of modulation was not robust for parametric communications, although it is robust for other types of communications.

### 4.3. Detection by Cross-Correlation of Modulation by Sine-Sweep

**Figure 8:**
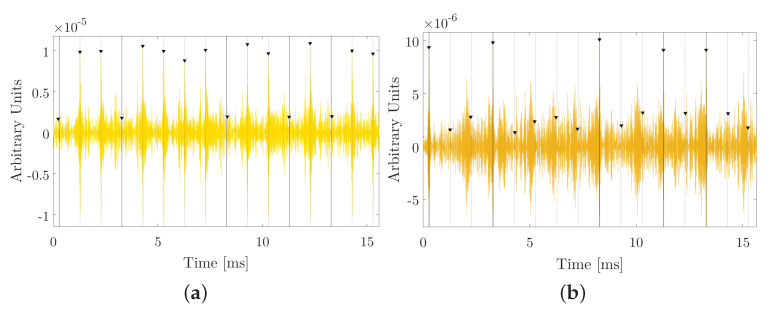
Frequency and correlation analysis. (**a**) Cross-correlation between the received signal and bit 1 upward from 10 until 50 kHz; (**b**) Cross-correlation between the received signal and bit 0 downward from 50 to 10 kHz at 1000 μs duration bits.

In a search for new modulation techniques that allow both temporarily locating each of bits 1 and 0, as well as discriminating between them, sine-sweep modulation is applied by applying parametric sweeps Figure 8. Given their robustness, these signals are being used recently as non-linear acoustic communication techniques [9,10]. Therefore, these signals will be analyzed by applying the 16-bit string to determine if the correlation detection is still appropriate for this type of signals.

Figure 8, it is observed that the peaks of the correlation are much narrower than in the case of the FSK modulations and, on the other hand, the correct bits with respect to the false ones are much clearer to discern than in the case of PSK modulation analyzed previously. The amplitudes for bit 1 and 0 are $0.1\times 10^{-4}$ and $0.095\times 10^{-4}$ (arbitrary units) respectively, and the amplitude values for the false bits are $0.018\times 10^{-4}$ and $0.023\times 10^{-4}$ (arbitrary units) for bit 1 and 0 respectively.

Amplitude ratio between the true bits 1 with respect to the false ones is 10.55, and amplitude quotient between true 0 bits with respect to false bits is 2.92. These data give us an estimate of the SNR for this modulation.

Among the modulations studied, the correlation peaks are detected very close to the expected times, considering the initial flight time where said signal is expected to be received. The behaviour of the correlation and the width of the resulting peaks has consequences for both detection and bit discrimination.

### 4.4. Bit Error

After the signals have been processed, the error bit (BER) of the three modulations used is analyzed through the following expression, BER (%) = (bits not detected/total bits) × 100

Regarding the FKS modulation, the measurements are presented in the Table 1, with their bit error. It is observed that the longer the duration of each bit, the less error there is in the detected bits, less than 20%. Even so, with the first group of signals sent (10, 30 kHz) and (10, 40 kHz) a BER of more than 50% was obtained. Regarding the value and the deviation of BER, it does not exceed ±7.

PSK modulation, it is observed in Table 2 that, between the signals and the duration times, there is no clear tendency when detecting the bit error. A BER of more than 50% is obtained in the 30 kHz signals at 67, 167 and 333 μs, 35 kHz at 57 and 429 μs, 40 kHz at 50 μs, and 50 kHz at 40 and 300 μs.

For the parametric sine-sweep modulation, it is observed, in Table 3, that the maximum error rate is for the sweep from 40 to 50 kHz with 90 μs of duration per bit and a BER of 58%. This is probably because of the short time time for each bit, they cannot be detected properly because they mix with the reflections from the bottom and surface of the pool. For the rest of the sweeps, the BER hovers around an average of 7%.

## 5. Influence of Propagation on an Acoustic Signal

**Figure 9:**
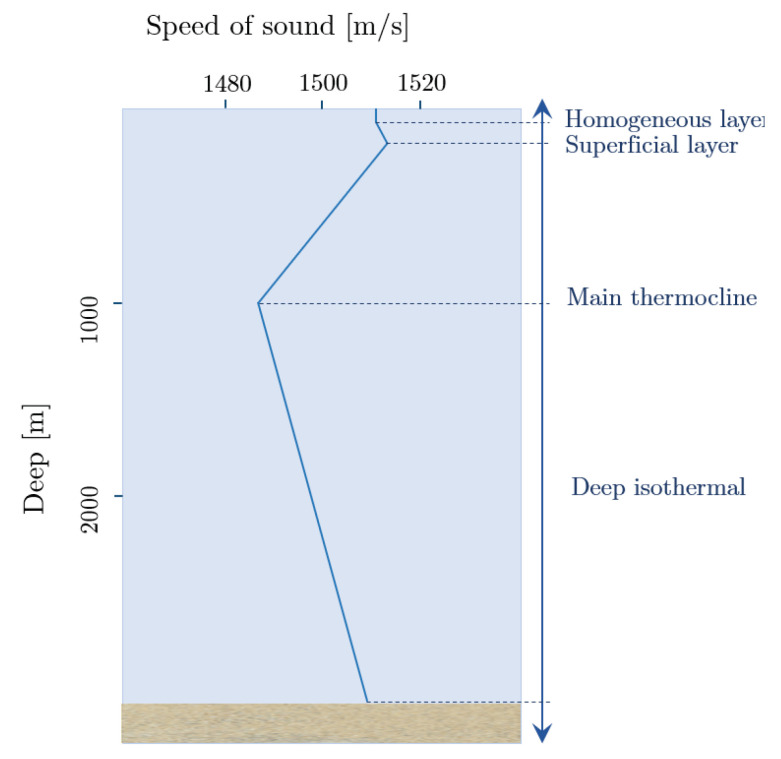
Typical profile of the speed of sound in sea water. Up to 300 m there is the surface layer, from 30 m and up to 1000 m the thermocline layer is included and from 1000 m the isothermal layer.

The generation of the multiple trajectories that a signal undergoes in the sea is conditioned by two effects: the reflection of the signal between the surface and the bottom and/or any object, and the refraction of sound in the water. The latter is a consequence of the variability of the speed of sound in water. The speed of sound depends on temperature, salinity and pressure, these, in turn, vary with depth and location. Signal rays tend to lean towards the region with the slowest propagation speed. Near the surface, velocity tends to be constant since temperature and pressure are also constant. In areas with warm temperatures, the temperature decreases as the depth begin to increase, however, the increase in pressure is not enough to compensate for the effect on the speed of sound. Thus, the speed of sound decreases in the region called the main thermocline. After a certain depth, the temperature reaches a constant level of ∼4°, and thereafter the speed of sound increases with depth (pressure). From this when a source emits a signal, this will follow a slightly different path due to the effects described above, and the receiver will receive not only the direct signal but each one the reflections. To more information details can be found in [15,16]. An example of the typical sound velocity profile in the marine environment is presented in Figure 9.

However, if we use the parametric effect, the effects of the reflections that can be generated in the middle will be much less, since they are highly directive sources at low frequencies. We can see this improvement in the next section. For this, a conventional source is compared and another source that emits a communication generated from said effect.

### Results of the Propagation Comparison between a Conventional and Parametric Source

**Figure 10:**
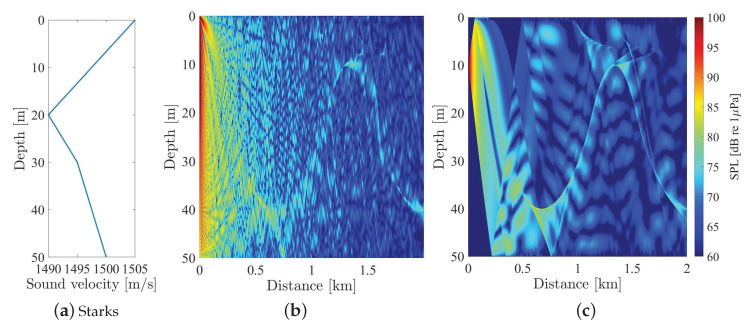
Behavior of a signal propagated in water with a conventional and a parametric transducer. (**a**) Sound speed profile in seawater; (**b**) acoustic fields generated with an omnidirectional source; (**c**) acoustic fields generated with an directional source one of ±10°.

The following Figure 10, shows two examples of the acoustic field generated by an acoustic emitter considering two different directivity beams. The simulation was implemented with Bellhop model for a constant bathymetric profile 50 m deep with which the source is 10 m deep, and a bed of sand has also been taken into account.

In Figure 10a, it is shown the variable propagation sound speed profile; in Figure 10b, the acoustic field with an omnidirectional source (directivity of ±90°); on the Figure 10c, the acoustic field with an directional source of ±10°. These directivities correspond, referring to previous studies by the authors on the behaviour of the parametric sound field [10], those generated by an Airmar P19 transducer emitting a parametric sweep between 4 and 40 kHz by modulating a 200 kHz carrier.

It is observed how the directivity influences the sound field. For the omnidirectional case, the mirror effect of the surface (Lloyd’s effect) is observed at close distances from the source, while for the directional source, this effect disappears, and the field presents more homogeneous. In both cases, a small acoustic channel is observed.

In shallow waters the profiles are more homogeneous (bottom sound channel or near constant). However, to show in a more emphatic way the implications of directivity in communication, we considered more illustrative to show the effects that could be produced in the case of an acoustic channel. In Figure 9 the interesting thing is to show the lower concentration of high SPL in the directive case than in the omnidirectional one. Furthermore, if we consider greater depths with the same acoustic channel, similar graphical results would be obtained.

**Figure 11:**
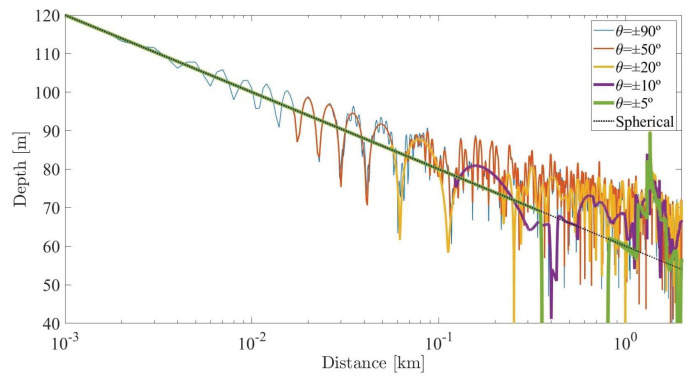
Sound pressure levels along a direction at the same depth of the acoustic source, considering different directivities.

Another way to analyze how directivity allows obtaining a field with less effect from multiple reflections is drawing the acoustic field along one direction, for example, in a horizontal direction at the same depth as the emitter, where the receiver of the underwater acoustic communication is placed. The Figure 11, shows this acoustic field for different source directivities. A line has been added that corresponds to a spherical spread without any reflection.

These analyzes will have a great impact on the design of underwater acoustic communications systems and on the evaluation of the improvement in available capacity from modulation methods.

## 6. Conclusions

This work presented a general study of some digital modulation techniques applied to a parametric generation focused on the field of transmission in underwater acoustic communications from a theoretical point of view, to later perform the analysis by the method of cross-correlation with signals experimentally measured. Based on this, the correlation was obtained for a 16-bit message (1 and 0) formed by the word “ok” with the FSK, PSK and the new modulation proposed, sine-sweep modulations, thus obtaining the maximum correlation peak for each of bit positions. In this sense, the three studied modulations were compared, obtaining better detection for sinusoidal sweep modulation, with fairly narrow and well-defined correlation peaks at each of the expected times.

The bit error that presents a minimum and independent percentage of the duration of each signal, corresponds to the modulation of parametric sweeps, mostly null and the others less than 7%.

The signal to noise ratio (SNR) allows us to establish a detection threshold based on the amplitudes of the correlation after the corresponding filtering. However, studies must be carried out that consider the variation of this bit error rate at greater distances, with greater environmental noise to correctly establish an optimal bit detection threshold.

These experiments have been carried out in a ideal laboratory conditions such as the Centro Tecnológico Naval y del Mar (CTN), in order to first of all whether this type of parametric modulations were suitable for submarine communications in shallow waters, where the influence of the reflections is greater. Secondly, we hope to present the studies in a subsequent publication that will allow us to obtain data regarding the range of the signal up to a distance of 15 km and measure these signals in a real marine environment, in order to take into account the influence of noise on our future experimental measurements.

With the use of parametric acoustic sources in shallow water, a significant performance is obtained in the reception of the signal since the beams that are emitted are highly directive. The improvement obtained with this type of technique is the reduction of the multiple paths in the signal, which would improve the interference between symbols.

## Figures and Tables

**Table 1:** Error Bit (BER) of the FSK modulation for a set of measured signals.

Frequencies [kHz]		Time [μs]	BER [%]		Frequencies [kHz]		Time [μs]	BER [%]
$f_{1}$	$f_{2}$				$f_{1}$	$f_{2}$		
10	30	500	59 ± 3		30	40	250	25 ± 0
10	40	1000	64 ± 5				500	19 ± 0
20	30	250	20 ± 7				1000	20 ± 7
		500	11 ± 3		30	50	100	66 ± 3
		1000	11 ± 5				167	45 ± 5
20	40	250	46 ± 3				250	69 ± 0
		500	15 ± 7				500	15 ± 7
		1000	8 ± 3				1000	10 ± 3
20	50	250	6 ± 3		40	50	50	69 ± 0
		500	0 ± 0				100	53 ± 3
		1000	0 ± 0				125	56 ± 4
30	40	100	20 ± 7				250	50 ± 0
		167	36 ± 5				1000	9 ± 7

**Table 2:** BER of the PSK for modulation for a set of measured signals.

Frequencies [kHz]	Time [μs]	BER [%]		Frequencies [kHz]	Time [μs]	BER [%]
10	500	25 ± 9		35	57	69 ± 0
	1000	28 ± 10			86	16 ± 8
15	200	41 ± 11			143	10 ± 4
	333	63 ± 5			286	11 ± 7
	667	69 ± 0			429	66 ± 3
	1000	9 ± 5		40	50	64 ± 5
20	250	29 ± 10			75	14 ± 11
	500	11 ± 7			125	14 ± 9
	750	10 ± 5			250	50 ± 10
25	80	0.6 ± 2			375	30 ± 12
	120	23 ± 9		45	44	34 ± 16
	200	29 ± 14			67	8 ± 8
	400	24 ± 10			111	8 ± 5
	600	34 ± 10			222	13 ± 10
30	67	69 ± 0			333	0 ± 0
	100	23 ± 13		50	40	62 ± 6
	167	68 ± 2			60	15 ± 14
	333	69 ± 0			100	0 ± 0
	500	35 ± 11			200	47 ± 12
					300	52 ± 12

**Table 3:** BER of the Sine-Sweep for modulation for a set of measured signals.

Frequencies [kHz]	Time [μs]	BER [%]		Frequencies [kHz]	Time [μs]	BER [%]
5 a 15	600	14 ± 3		10 a 40	160	0 ± 0
	1000	0 ± 0			320	0 ± 0
5 a 25	267	1 ± 3			480	0 ± 0
	400	0 ± 0		10 a 50	134	1 ± 3
	533	0 ± 0			267	0 ± 0
5 a 35	200	1 ± 3			400	1 ± 3
	400	0 ± 0			1000	0 ± 0
	600	0 ± 0		20 a 30	160	15 ± 7
5 a 45	160	6 ± 8			320	0 ± 0
	320	1 ± 3			480	0 ± 0
	480	0 ± 0		20 a 40	134	0 ± 0
	147	8 ± 5			267	0 ± 0
5 a 50	291	3 ± 3			402	0 ± 0
	438	0 ± 0		20 a 50	115	13 ± 8
4 a 40	500	0 ± 0			229	0 ± 0
	1000	0 ± 0			343	0 ± 0
10 a 20	267	5 ± 3		30 a 40	115	15 ± 14
	534	0 ± 0			229	6 ± 0
	801	0 ± 0			343	0 ± 0
10 a 30	160	4 ± 6		30 a 50	100	18 ± 8
	200	0 ± 0			200	0 ± 0
	320	10 ± 7			600	0 ± 0
	400	0 ± 0		40 a 50	90	58 ± 12
	600	0 ± 0			178	10 ± 3
					267	0 ± 0
